# Engineered Cas9 variant tools expand targeting scope of genome and base editing in rice

**DOI:** 10.1111/pbi.13293

**Published:** 2019-11-20

**Authors:** Dongchang Zeng, Xitao Li, Jiafa Huang, Yangyang Li, Shuqiong Cai, Weizhi Yu, Yingli Li, Yinpin Huang, Xianrong Xie, Qi Gong, Jiantao Tan, Zhiye Zheng, Menghui Guo, Yao‐Guang Liu, Qinlong Zhu

**Affiliations:** ^1^ State Key Laboratory for Conservation and Utilization of Subtropical Agro‐Bioresources Guangzhou China; ^2^ College of Life Sciences South China Agricultural University Guangzhou China; ^3^ Guangdong Laboratory for Lingnan Modern Agriculture Guangzhou China; ^4^ Chongqing Engineering Research Center for Floriculture College of Horticulture and Landscape Southwest University Chongqing China

**Keywords:** genome editing, base editing, xCas9, Cas9‐NG, eCas9‐NG, rice

CRISPR/Cas9 systems have been widely used in functional genomics and crop genetic improvement, but the protospacer adjacent motif (PAM) sequence NGG of *Streptococcus pyogenes* Cas9 (SpCas9) limits its targeting scope. To expand the targetable genomic loci, several other Cas proteins and Cas9 variants with different PAM specificities have been developed (Li *et al.*, 2019), such as Cpf1 (Cas12a) with T‐rich PAM, and SpCas9 VQR and VRER variants with non‐canonical NGA and NGCA PAM sequence, respectively. Recently, two engineered SpCas9 variants, xCas9 3.7 and Cas9‐NG, expand the PAM recognition site to NG and show function efficiency in mammalian cells (Hu *et al.*, 2018; Nishimasu *et al.*, 2018). Although a few tests of the two variants have been reported in plants (Ge *et al.*, 2019; Li *et al.*, 2019), there are still not enough comparative data on their efficiencies on genome and base editing in plants. Furthermore, to reduce the possible off‐target effects, it is necessary to develop a new Cas9 variant with both enhanced specificity (eCas9) and altered PAM site such as NG. The eCas9 variant has neutralization of positive charges in the nt‐groove that can remarkably decrease off‐target (Slaymaker *et al.*, 2016). In this study, we developed a serial of variants including a new eCas9‐NG and investigated the editing activities of those variants (xCas9, Cas9‐NG and eCas9‐NG) with expended target scope in genome editing, cytosine base editors including CBE4, xCas9n‐CBE, Cas9n‐NG‐CBE and eCas9n‐NG‐CBE, adenine base editors including ABE7.10, xCas9n‐ABE, Cas9n‐NG‐ABE and eCas9n‐NG‐ABE in transgenic rice.

We first generated the xCas9 and Cas9‐NG (Figure 1a) based on our previous rice codon‐optimized SpCas9 (Ma *et al.*, 2015) referring to the xCas9 3.7 (Hu *et al.*, 2018) and the Cas9‐NG variants (Nishimasu *et al.*, 2018). Then, the eCas9‐NG variant with K848A/K1003A/R1060A mutations (Slaymaker *et al.*, 2016) was produced using the Cas9‐NG (Figure 1a). We selected four target sites with TGN PAMs in *OsWaxy* (encoding starch synthase enzyme I) to test their genome‐editing activities, using the wild‐type SpCas9 as a control. We prepared four multiplex genome‐editing constructs for rice transformation, each having four sgRNA expression cassettes targeting the TGN target sites, respectively. The results showed the Cas9‐NG had the wider targeting scope and different levels of editing activities at non‐canonical TGA, TGT and TGC PAM sites (9.1%–45.5%) (Figure 1b), which were similar to the previous reports (Zhong *et al.*, 2019). Compared with Cas9‐NG, the new eCas9‐NG showed lower editing efficiency at non‐canonical target sites with TGA (5.5%) and TGC (8.3%) (Figure 1b), suggesting that the higher specificity of eCas9‐NG may affect its editing efficiency. The xCas9 only showed low editing activities (6.1%) at TGG PAM (Figure 1b), which was consistent with the previous findings that the editing efficiencies of xCas9 were low and mainly detected at the NGG PAM in *Arabidopsis* and rice (Ge *et al.*, 2019; Li *et al.*, 2019). However, the editing efficiencies of Cas9‐NG (27.3%) and xCas9 (6.1%) at canonical TGG PAM were significantly reduced compared with that of Cas9 (76.5%; Figure 1b). These results indicate that the Cas9‐NG and eCas9‐NG could recognize non‐canonical PAMs in rice, and their editing activities are better than xCas9. Both variants could generate loss‐of‐function mutants of the target gene, such as an *OsWaxy*‐knockout mutant by eCas9‐NG (Figure 1c).

**Figure 1:**
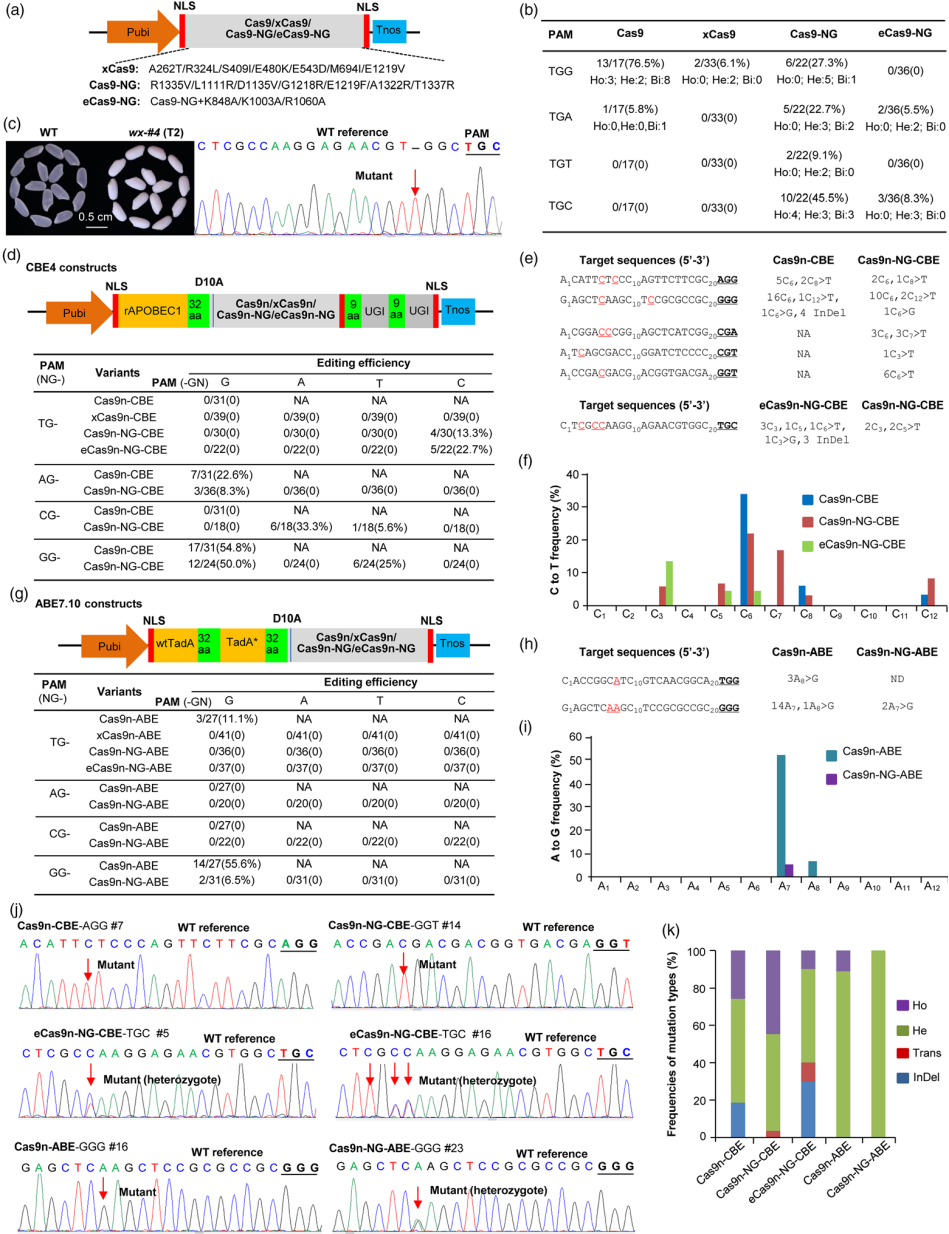
The Cas9 variant‐based genome‐editing tools expanding the target scope in rice. (a) Diagrams of genome editing using four Cas9 variants. NLS: nuclear localization signal. (b) Targeted mutation efficiencies and patterns by four Cas9 variants at different PAMs. Ho: homozygous mutation; He: heterozygous mutation; Bi: biallelic mutation. (c) The phenotype of glutinous rice grains (with white colour endosperm) by knocking out *OsWaxy* with eCas9‐NG. Sequencing showed a ‘T’ insertion (arrowed) in the coding region of *OsWaxy*. (d) Diagrams of four CBE4 editors and their cytosine base‐editing efficiencies. NA: not available. (e, f) Detailed target sequences, edited positions and editing windows of C to T editing by the CBE4 editors. (g) Diagrams of four ABE7.10 editors and their adenine base‐editing efficiencies. (h, i) Detailed target sequences, edited positions and editing windows of A to G editing by the ABE7.10 editors. (j) Sequencing chromatogram of some base‐editing examples by the CBE4 or ABE7.10 editors in transgenic rice. (k) The frequencies of various mutation types induced by CBE4 or ABE7.10 editors in all mutants. Trans: transversion; InDel: insertion/deletion.

For traditional CBE and ABE editors, their target windows are substantially limited by recognizing NGG PAM (Mishra *et al.*, 2019). Therefore, we tested the potential of these variants to expand the target region of base editing in the rice genome. We fused the rice codon‐optimized rAPOBEC and double UGIs to the N‐terminus and C‐terminus of the nickase variants (D10A) of xCas9, Cas9‐NG and eCas9‐NG, respectively, to generate four CBE4 base editors (Figure 1d). To compare their CBE efficiency, we selected different target sites with PAMs of TGN and AGN in *OsWaxy*, CGN in *OsEUI1* (*Elongated Uppermost Internode 1*) and GGN in *OsCKX2* (encoding a cytokinin oxidase/dehydrogenase) to construct multiple‐targeting vectors for rice transformation. Sequencing analysis of the transformants showed that only Cas9n‐NG‐CBE and eCas9n‐NG‐CBE produced the expected C‐T conversion at the TGC PAM with efficiency of 13.3% and 22.7%, respectively, but no base‐editing activity was detected for target sites with TGG, TGA and TGT PAMs (Figure 1d,j). In addition, insertion/deletion (InDel) mutation was detected in these sites targeted by Cas9n‐CBE and eCas9n‐NG‐CBE but not by Cas9n‐NG‐CBE (Figure 1e). For other tested PAMs, Cas9n‐NG‐CBE showed different levels of C‐T substitution at canonical PAMs of GGG (50%) and AGG (8.3%), and non‐canonical PAMs of CGA (33.3%), GGT (25%) and CGT (5.6%), respectively (Figure 1d).

For all tested PAMs, the editing windows of Cas9n‐NG‐CBE were mainly distributed in C_3_ ~ C_8_ and mainly at C_6_ position (Figure 1d–f), which was consistent with the report in rice (Wang *et al.*, 2019). Compared with other CBE editors, such as Cas9n‐NG variants fused with activation‐induced deaminase PmCDA1 (Zhong *et al.*, 2019) or hAID (Hua *et al.*, 2019), our Cas9n‐NG‐CBE showed slightly higher base‐editing efficiency than those variants. The Cas9n‐CBE had the C‐T conversion at the canonical GGG (54.8%) and AGG (22.6%) PAMs (Figure 1d), and the editing window was mainly in C_6_ position (Figure 1d–f). However, xCas9n‐CBE did not detect base‐editing activity at TGN PAMs, suggesting it work ineffectively in rice, which is similar to the observations of low efficiencies of xCas9n‐CBE in rice (Hua *et al.*, 2019; Li *et al.*, 2019). The possible reasons for the inefficiency of xCas9 and xCas9n‐CBE are that their mutations (R324L, S409I and M694I) may affect the recognition and binding of DNA‐sgRNA (Hu *et al.*, 2018; Li *et al.*, 2019). These results indicate that the Cas9n‐NG‐CBE worked in rice at endogenous NG sites with a broad range of PAM sequences and had high base‐editing activity at canonical NGG PAM.

To expand the target scope of ABE editors, we fused the wtTadA and evolutionary TadA (TadA*) of ABE7.10 base editor (Mishra *et al.*, 2019) to the N‐terminus of above Cas9 nickase variants (D10A), to generate Cas9n‐ABE, xCas9n‐ABE, Cas9n‐NG‐ABE and eCas9n‐NG‐ABE, respectively (Figure 1g). We selected the same editing sites as above, and the results showed these ABE7.10 editors barely produced base editing at non‐canonical PAM‐containing sites, except for Cas9n‐ABE at sites with canonical PAMs of GGG (55.6%) and TGG (11.1%), and for Cas9n‐NG‐ABE at sites with GGG (6.5%), with the editing window mainly at A_7_ position (Figure 1h–j). The similar results in rice were observed previously that ABE‐NG has very low editing activity at sites with PAMs of CGG (2.6%), AGC (2%) and CGT (2.9%), and no adenine base editing was detected using the xCas9n variant (Hua *et al.*, 2019), suggesting that ABE 7.10 base editor is less efficient in rice, and further efforts are also required to test other adenine base editors. These CBE4 and ABE7.10 editors mentioned above produced different frequencies and mutation types, of which Cas9n‐NG‐CBE induced highest homozygous editing rate (44.8%; Figure 1k).

Due to more relaxed NG PAM, we detected these Cas9 variants' off‐target editing possibility for each on‐target site with TGN PAMs. The potential off‐target sites were selected using CRISPR‐GE (http://skl.scau.edu.cn/) for sequencing. As expected, we did not find off‐target effects of the candidate off‐target sites in editing plants by eCas9‐NG. However, no off‐target effects were also detected at the potential sites using Cas9, xCas9 and Cas9‐NG, respectively. These results suggested that these Cas9 variants have a certain degree of specificity, and the specificity of eCas9‐NG is also necessary to evaluate at more target sites.

In conclusion, our results indicate that the Cas9‐NG and eCas9‐NG variants enable more efficient genome editing and targeted C‐T single nucleotide substitutions with extended non‐canonical PAMs than those of xCas9 in rice. The Cas9‐NG variant is more suitable for genome engineering in rice. The versatile Cas9 variant tools for genome engineering expand the target scope in rice and possible in other plants, thus will facilitate plant functional genome and crop genetic improvement.

## Conflict of interest

The authors declare no conflict of interest.

## Author contributions

Y.‐G.L. and Q.Z. designed the studies. D.Z., X.L., J.H., Y.L., S.C., W.Y., Y.L., Y.H., X.X., Q.G., J.T., Z.Z. and M.G. performed experiments. Q.Z., Y.‐G.L. and D.Z. wrote the paper.
